# A Network Pharmacology Approach to Uncover the Mechanisms of Shen-Qi-Di-Huang Decoction against Diabetic Nephropathy

**DOI:** 10.1155/2018/7043402

**Published:** 2018-11-01

**Authors:** Sha Di, Lin Han, Qing Wang, Xinkui Liu, Yingying Yang, Fan Li, Linhua Zhao, Xiaolin Tong

**Affiliations:** ^1^Department of Endocrinology, Guang'anmen Hospital, China Academy of Chinese Medical Sciences, Beijing 100054, China; ^2^Department of Clinical Chinese Pharmacy, School of Chinese Materia Medica, Beijing University of Chinese Medicine, Beijing 100102, China; ^3^Shenzhen Hospital of Guangzhou University of Chinese Medicine, Shenzhen 518034, China

## Abstract

Shen-Qi-Di-Huang decoction (SQDHD), a well-known herbal formula from China, has been widely used in the treatment of diabetic nephropathy (DN). However, the pharmacological mechanisms of SQDHD have not been entirely elucidated. At first, we conducted a comprehensive literature search to identify the active constituents of SQDHD, determined their corresponding targets, and obtained known DN targets from several databases. A protein-protein interaction network was then built to explore the complex relations between SQDHD targets and those known to treat DN. Following the topological feature screening of each node in the network, 400 major targets of SQDHD were obtained. The pathway enrichment analysis results acquired from DAVID showed that the significant bioprocesses and pathways include oxidative stress, response to glucose, regulation of blood pressure, regulation of cell proliferation, cytokine-mediated signaling pathway, and the apoptotic signaling pathway. More interestingly, five key targets of SQDHD, named AKT1, AR, CTNNB1, EGFR, and ESR1, were significant in the regulation of the above bioprocesses and pathways. This study partially verified and predicted the pharmacological and molecular mechanisms of SQDHD on DN from a holistic perspective. This has laid the foundation for further experimental research and has expanded the rational application of SQDHD in clinical practice.

## 1. Introduction

Diabetic nephropathy (DN), a complex and multifaceted condition, is one of the main microvascular complications of diabetes mellitus, especially type 2 diabetes mellitus (T2DM) [1]. T2DM is an important cause of kidney failure, which presents the risk of development of hypertension. In 2010, 6.4% of the world's population was diagnosed with diabetes mellitus, and this value is expected to increase to 7.7% in 2030, in other words, from 285 million to 439 million adults [2]. DN is distinguished by the elevated albumin excretion rate and/or the transient increased glomerular filtration rate (GFR) [3]. The earliest sign of DN is microalbuminuria (>30 mg/day), which develops into macroalbuminuria (>300 mg/day) and decreased GFR, eventually leading to end-stage-renal disease (ESRD) [4, 5]. The pathogenesis of DN has been associated with oxidative stress and inflammation caused by chronic high blood glucose [6–8], glucose metabolic disorder [9], hemodynamics, and hemorheology anomalies [10]. The current standard therapy includes intensive treatment and control of hyperglycemia and blood pressure. A blockade of the renin-angiotensin system (RAS) is also associated [11]; however, RAS combination therapy cannot prevent the progression of DN and is linked to an elevated rate of severe adverse events. Novel agents have shown controversial results or side effects [12] which makes it important to develop more efficient treatment to cure DN and reduce side effects.

Traditional Chinese Medicine (TCM) is widely propagated and used in more than 100 countries across the world owing to its satisfactory clinical efficacy [13]. SQDHD was documented in Shen Shi Zun Sheng Shu, which was written by Shen Jinao in 1773 during the Qing Dynasty. SQDHD contains eight Chinese herbs, including* Codonopsis Radix *(Dang Shen [DS]),* Hedysarum Multijugum Maxim.* (Huang Qi [HQ]),* dried Radix Rehmannia *(Sheng Di Huang [SDH]),* Rhizoma Dioscoreae *(Shan Yao [SY]),* Cornus Officinalis Sieb. Et Zucc.* (Shan Zhu Yu [SZY]),* Cortex Moutan *(Mu Dan Pi [MDP]),* Alisma Orientale (Sam.) Juz.* (Ze Xie [ZX]), and* Poria Cocos(Schw.) Wolf.* (Fu Ling [FL]). Liuwei dihuang pill (LDP), including* Cornus Officinalis Sieb. Et Zucc., Cortex Moutan, Rhizoma Dioscoreae, Poria Cocos(Schw.) Wolf., Alisma Orientale (Sam.) Juz., *and Radix Rehmanniae Praeparata, inhibited erythrocyte aldose reductase activity and lowered urinary albumin excretion rate and beta2-MG in the blood and urine in the treated group compared to those in the control group [14]. LDP can decrease multiple pathways including TGF-*β*/SMADS, MAPK, and NF-*κ*B signaling to prevent the progress of renal fibrosis and defend glomerular mesangial cells [15]. Astragaloside IV (ASI), active component in* Hedysarum Multijugum Maxim.,* could inhibit high glucose-induced cell apoptosis and decrease TGF-*β*1 and the activity of p38 in the MAPK pathway [16]. Dried Rehmanniae Radix reduced glucose, urea nitrogen, 5-hydroxymethylfurfural, and thiobarbituric acid- (TBA-) reactive substance levels in DN rats [17]. Moutan Cortex could significantly decrease blood glucose, serum creatinine, and urine protein in DN rats and reduce transforming growth factor beta 2 (TGF-*β*2) in renal tissue [18]. Therefore, SQDHD might exhibit substantial effect on DN. As SQDHD includes many chemical compounds and adjusts a variety of targets, the pharmacological mechanisms require a complete clarification, which has been a challenge.

**Figure 1:**
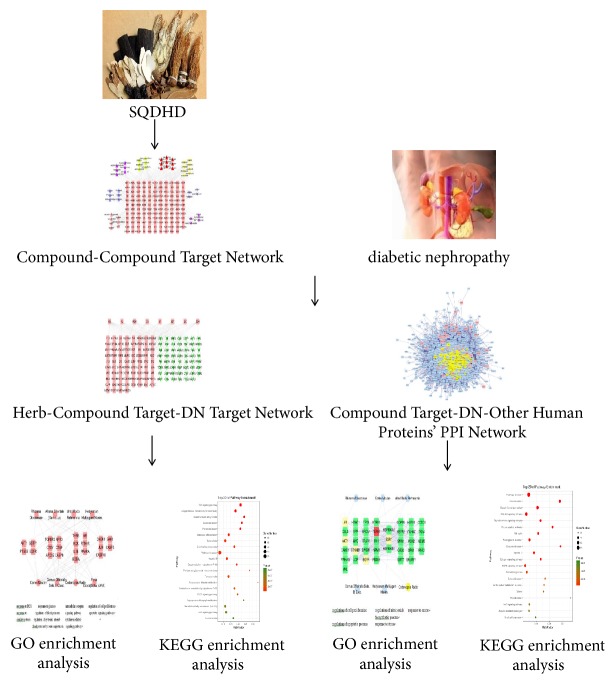
Workflow for SQDHD against diabetic nephropathy.

Network pharmacology, put forward by Hopkins in 2007, is used to elucidate the drugs effect on multiple targets [19]. Network pharmacology can build networks to reflect and clarify the interactive relationship between multiple components, multiple targets, multiple pathways, and complex diseases. It is also capable of interpreting the mechanisms of functional drugs based on the network built on public databases or available data through earlier researches. Network pharmacology can reconstruct a “drug target disease” network prediction model [20, 21]. TCM herbal formulas treat and prevent disease, and is composed of multicomponents and multitargets; thus, its mechanisms can be investigated by network pharmacology. The study was aimed at elucidating the pharmacological mechanisms of SQDHD in DN treatment using the comprehensive network pharmacology. The workflow of network pharmacology-based study of SQDHD against diabetic nephropathy was exhibited in Figure 1.

## 2. Material and Methods

### 2.1. Data Preparation

#### 2.1.1. Composite Compounds of Each Herb in SQDHD

Seventy-seven active compounds from the eight herbs found in SQDHD were screened from domestic and foreign literatures, and the Traditional Chinese Medicine Systems Pharmacology Database [22] (TCMSP, http://lsp.nwu.edu.cn/tcmsp.php). TCMSP is a unique pharmacology platform for Chinese herbal medicines. Eleven compounds in Codonopsis Radix [23–29], 7 in Poria Cocos (Schw.) Wolf [30, 31], 11 in Hedysarum Multijugum Maxim [32–34], 9 in Cortex Moutan [35, 36], 12 in Rhizoma Dioscoreae [37–40], 12 in Cornus Officinalis Sieb. Et Zucc [41–51], 5 in Radix Rehmanniae [52, 53], and ten in Alisma Orientale (Sam.) Juz [54–56] were collected as well as the Canonical SMILES of all active compounds from TCMSP. The details are described in Table S1. Three-dimensional chemical structure data of active compounds was found and exported from PubChem [57] (https://pubchem.ncbi.nlm.nih.gov/).

#### 2.1.2. Compound Targets for Each Herb in SQDHD

The compound targets of each herb found in SQDHD were collected from Stitch [58] (http://stitch.embl.de/, ver. 5.0), by inputting the Canonical SMILES into SMILES string(s), with the organism selected as “Homo sapiens” and a confidence score >0.4. The compound targets having no relationship with the compounds were deleted. Stitch is a resource to explore interactions between chemicals and proteins. Input 3D structure of active compounds into PharmMapper [59] (http://lilab.ecust.edu.cn/pharmmapper/index.php, Updated on Nov 27, 2017), a freely accessed web-server designed to identify potential target candidates for given probe small molecules (drugs, natural products, or other newly discovered compounds with binding targets unidentified) using the pharmacophore mapping approach. We used UniProtKB [60] (http://www.uniprot.org/) to obtain the standard compound targets' names. This database provides the scientific community with a comprehensive, high-quality, and freely accessible resource of protein sequence and functional information. The protein names were entered into UniProtKB, with the organism selected as “Homo sapiens,” prior to the retrieval of the official symbol. We therefore obtained the compound targets for each herb in SQDHD. The details are described in Table S2.

#### 2.1.3. DN Targets

The DN targets were obtained from the Therapeutic Target Database [61] (TTD, https://db.idrblab.org/ttd/, Updated in Sep. 10, 2017), Online Mendelian Inheritance in Man® [62] (OMIM, http://omim.org/about/), DrugBank [63] (https://www.drugbank.ca/, Updated in Dec. 20, 2017, ver. 5.0.11), and the Genetic Association Database (GAD, https://geneticassociationdb.nih.gov/). TTD provides information regarding the known and explored therapeutic protein and nucleic acid targets, the targeted disease, pathway information and the corresponding drugs directed at each of the targets. Seventeen targets for DN were collected and the details are described in Table S3. OMIM is a comprehensive, authoritative compendium of human genes and genetic phenotypes that is freely available and updated daily. The full-text, referenced overviews in OMIM contain information on all known mendelian disorders and over 15,000 genes. The DrugBank database is a comprehensive, freely accessible, online database containing information on drugs and drug targets. The GAD is a database of genetic associational data from complex diseases and disorders. After serving the scientific community for more than 10 years, GAD has been retired and all data is “frozen” as of 09/01/2014. However, all GAD data as of 08/18/2014 will continue to be available.

#### 2.1.4. Protein-Protein Interaction Data

All protein-protein interaction (PPI) data were derived from STRING [64] (https://string-db.org/, ver. 10.5), and the organism was selected as “Homo sapiens” and a confidence score >0.7. The STRING database, an update on the online database, collects and presents known and predicted PPI with a confidence score and accessory information. The score represents the interaction confidence of the protein, which has a positive relationship [65].

### 2.2. Network Construction

#### 2.2.1. Network Construction Method

The network construction was performed as follows: (1) the active compounds-active compounds target network of SQDHD was built, (2) the herb-compound target-DN target network was built via linking the eight SQDHD herbs with compound targets of each herb, and DN targets, and (3) the compound targets-DN targets-other human proteins network was built.

We used the network visualization software Cytoscape [66] (http://cytoscape.org/, ver. 3.5.0) to build all networks. Cytoscape is an open source software platform for complex network analysis and visualization. It visualizes molecular interaction networks and biological pathways and integrating these networks with annotations, gene expression profiles, and other state data.

#### 2.2.2. Network Topological Feature Set Definition

We used three indices to evaluate every node in the network.

The three indices contain degree, betweenness, and closeness of nodes. Degree represents the number of edges between a node and another node in the network [67]. Betweenness evaluates the participation of a node in the shortest parts of the network and reflects the ability of nodes to deal with the rate of information flow in the network as well [68]. Closeness is the inverse of the sum of the distance from a node to other nodes. The three indices play an important role in the network, and the level of the indices has a positive association with the importance of node in the network [69].

### 2.3. Gene Ontology Enrichment Analysis

The Database for Annotation, Visualization, and Integrated Discovery [70] (DAVID Bioinformatics Resources, https://david.ncifcrf.gov/, ver. 6.8) was utilized for the Gene Ontology (GO) enrichment analysis. DAVID provides a comprehensive set of functional annotation tools so that investigators can understand the biological meaning behind large lists of genes.

## 3. Results and Discussion

### 3.1. Compound-Compound Target Network Analysis

**Figure 2:**
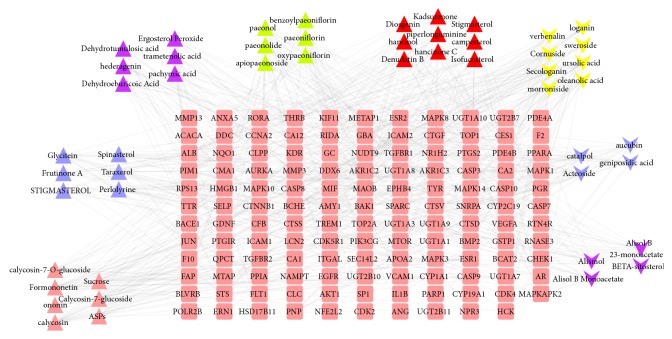
Compound-compound target network (pink rounded rectangles represent compound targets; pink triangles, blue triangles, purple triangles, green triangles, red triangles, yellow v, blue v, and purple v represent compounds of Hedysarum Multijugum Maxim., Codonopsis Radix, Poria Cocos(Schw.) Wolf., Cortex Moutan, Rhizoma Dioscoreae, Cornus Officinalis Sieb. Et Zucc., dried Radix Rehmannia, and Alisma Orientale (Sam.) Juz., respectively).

The network consists of 181 nodes (50 compounds in SQDHD and 131 compound targets) and 551 edges, as shown in Figure 2. The network suggests that many compound targets can be adjusted by multiple compounds, while the number of compound targets such as VEGF-A, CLC, AKT1, and PIK3CG can be regulated by just one compound. PIM1 can be regulated by 24 compounds, and STS, MAOB, CA2, and BMP2 can be regulated by 20 compounds, which may be vital compound targets in SQDHD. From the network, we can make a rough observation of the relationship between active compounds and compound targets.

### 3.2. Herb-Compound Target-DN Target Network Analysis

**Figure 3:**
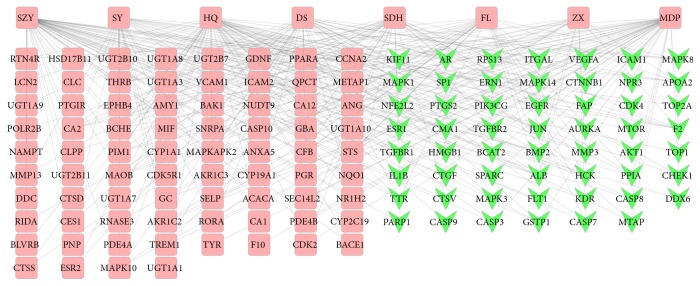
Herb-Compound Target-DN Target Network (the eight rounded rectangles at the top of the figure represent herbs, the ones below on the left (pink rounded rectangles) represent compound, and green v represents compound targets/DN targets).

This network was built to show the relationship between eight herbs, compound targets, and DN targets. In Figure 3, the network consists of 139 nodes (eight herbs, 76 compound targets, and 55 compound targets/DN targets) and 300 edges. The network shows that the compound targets are also controlled by drug targets (DN targets), which suggests that drugs may indirectly regulate disease-related proteins, while SQDHD can directly affect these proteins. SQDHD may also indirectly affect drug targets by controlling related proteins (compound targets). We also found SZY (58) having the highest connection with other nodes, followed by HQ, MDF (56, 45). This suggests their significance in the network.

**Figure 4:**
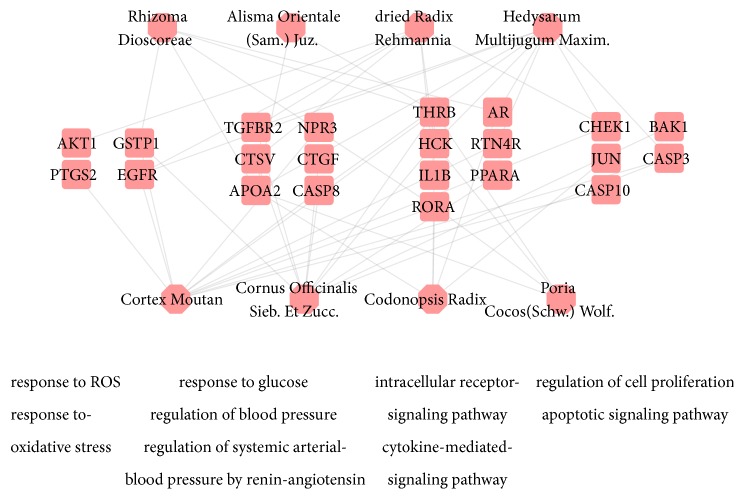
According to the associated biological processes or pathways, compound targets of SQDHD and DN targets are related to various molecular mechanisms of DN (The pink octagons and pink rounded rectangles represent the eight herbs in SQDHD and targets, respectively).

In Figure 4, according to the GO enrichment analysis, compound targets, DN targets, and compound targets/DN targets are significantly associated with response to oxidative stress (GO:0006979; Fold Enrichment = 4.45;*P* =0.011), response to reactive oxygen species (GO:0000302; Fold Enrichment = 8.36;*P* =0.011), response to glucose (GO: 0009749; Fold Enrichment = 10.79;*P*< 0.001), regulation of systemic arterial blood pressure by renin-angiotensin (GO:0003081; Fold Enrichment = 40.76;*P*= 0.0021), regulation of blood pressure (GO:0008217; Fold Enrichment = 13.79;*P*< 0.001), regulation of cell proliferation (GO:0042127; Fold Enrichment = 4.41;*P*< 0.001), cytokine-mediated signaling pathway (GO:0019221; Fold Enrichment = 4.36;*P*= 0.0054), apoptotic signaling pathway (GO:0097190; Fold Enrichment = 5.74;*P* =0.011), and intracellular receptor signaling pathway (GO:0030522; Fold Enrichment = 8.58;*P* =0.011). The details are described in Table S4.

In the network (Figure 4), there are numerous DN-related biological processes, including response to oxidative stress (GO:0006979), response to glucose (GO: 0009749), regulation of blood pressure (GO:0008217), regulation of cell proliferation (GO:0042127), and cytokine-mediated signaling pathway (GO:0019221). These processes are the potential mechanisms involved in the treatment of DN. Some literature has reported several biological processes obtained using DAVID. Hypertension and hyperglycemia play vital roles in the processes of DN. Cytokines (vascular endothelial growth factor (VEGF), CC chemokine receptor 2 (CCR2), TGF-*β*, protein kinase C (PKC)), oxidative stress, and inflammation are also key elements in the processes of DN. Currently, standard treatments for DN include controlling hyperglycemia and blood pressure by inhibiting RAS [13, 71, 72]. The RAS may increase renovascular resistance and intraglomerular pressure, leading to renal damage [73], hyperglycemia, rise in matrix production, or glycation of matrix proteins. These processes subsequently lead to hyperglycemia, which results in vessel injury followed by kidney mesangial expansion and injury [13].

Intracellular reactive oxygen species (ROS) increases the secretion by mitochondria in glucose stimulation. This occurs through five main pathways including the pathogenesis of complications: polyol pathway flux, increased formation of advanced glycation end products (AGEs), increased expression of the receptor for AGEs and its activating ligands, activation of PKC isoforms, and overactive hexosamine pathway. Elevated ROS leads to ischemia and defective angiogenesis and then activates many proinflammatory pathways [74–76]. NO can negatively control mitochondrial oxidative metabolism, by binding to cytochrome c oxidase. ROS directly decreases eNOS activity [77]. The chronic hyperglycemic milieu markedly increases AGEs in both the cellular and extracellular compartments in various tissues [78, 79]. AGEs binding to the receptor for AGEs (RAGE) increase ROS production. ROS can regulate PKC pathway in mesangial cells and increase TGF-*β* which is associated with kidney fibrosis. Meanwhile, ROS can also activate NF-*κ*B in mesangial cells, leading to inflammatory response. ROS results in glomerular mesangial expansion and renal tubulointerstitial fibrosis, via disturbing cell function, and signal transduction cascades [9, 80, 81]. Insulin-dependent diabetic BB rats and NOD mouse had elevated expression of TGF-*β* in the kidney, and plasma and urine of DN patients had higher VEGF. This suggests the urinary VEGF might be used as a sensitive marker of DN and for predicting disease progression [82, 83].

**Figure 5:**
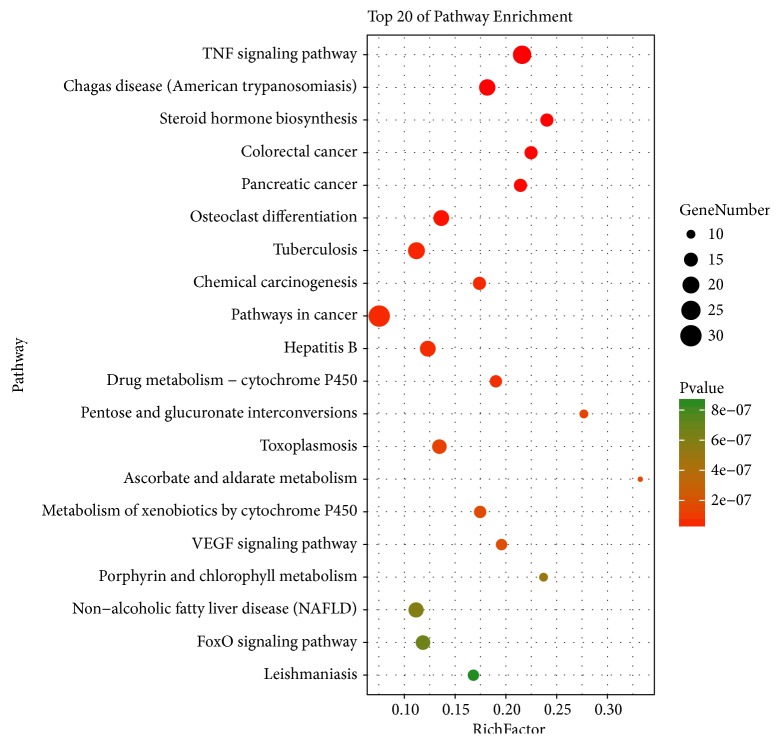
Differentially expressed gene PATHWAY enrichment point diagram (the vertical axis represents the pathway name, the horizontal axis represents the Rich factor, the size of the dot indicates the number of genes expressed in the pathway, and the color of the dot corresponds to the different Qvalue range).

In Figure 5, according to KEGG enrichment analysis, compound targets, DN targets, and compound targets/DN targets are significantly associated with TNF signaling pathway, Chagas disease, steroid hormone biosynthesis, tuberculosis, VEGF signaling pathway and others. The most enriched pathways involving significant differential expression are TNF signaling pathway [84], steroid hormone biosynthesis [85, 86], colorectal cancer [87], pancreatic cancer, glucuronate interconversions [88], and VEGF signaling pathway [89]. The details are described in Table S5.

### 3.3. Compound Target-DN-Other Human Proteins' PPI Network Analysis

**Figure 6:**
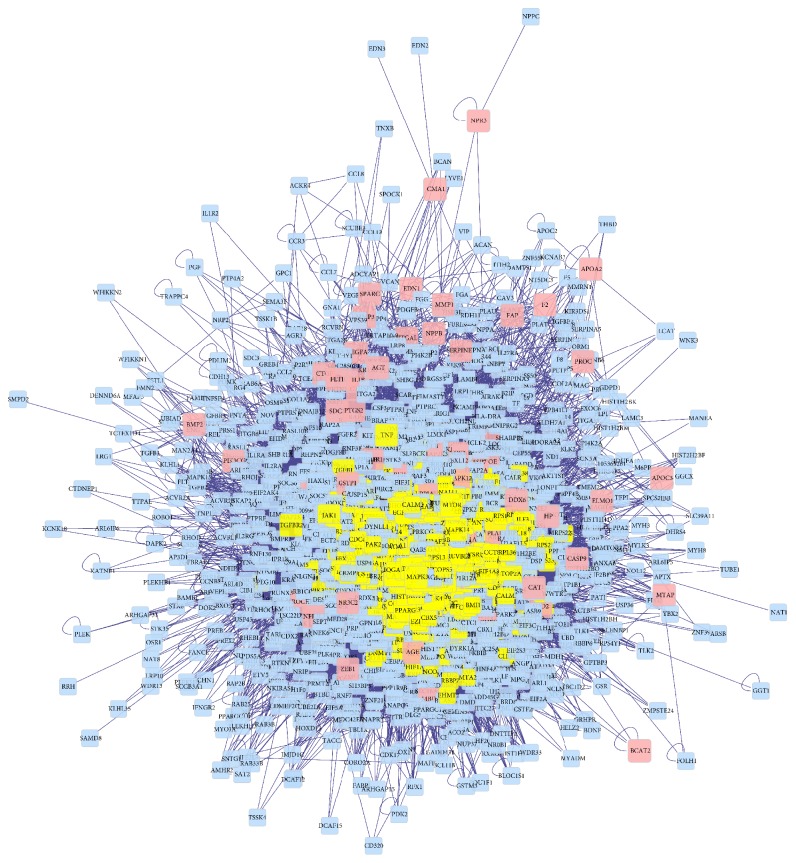
Compound Target-DN-Other Human Proteins' PPI Network (pink nodes, yellow nodes, blue nodes represent compound targets and DN targets, the significant targets, and other human proteins interacting with the compound or DN targets, respectively).

**Figure 7:**
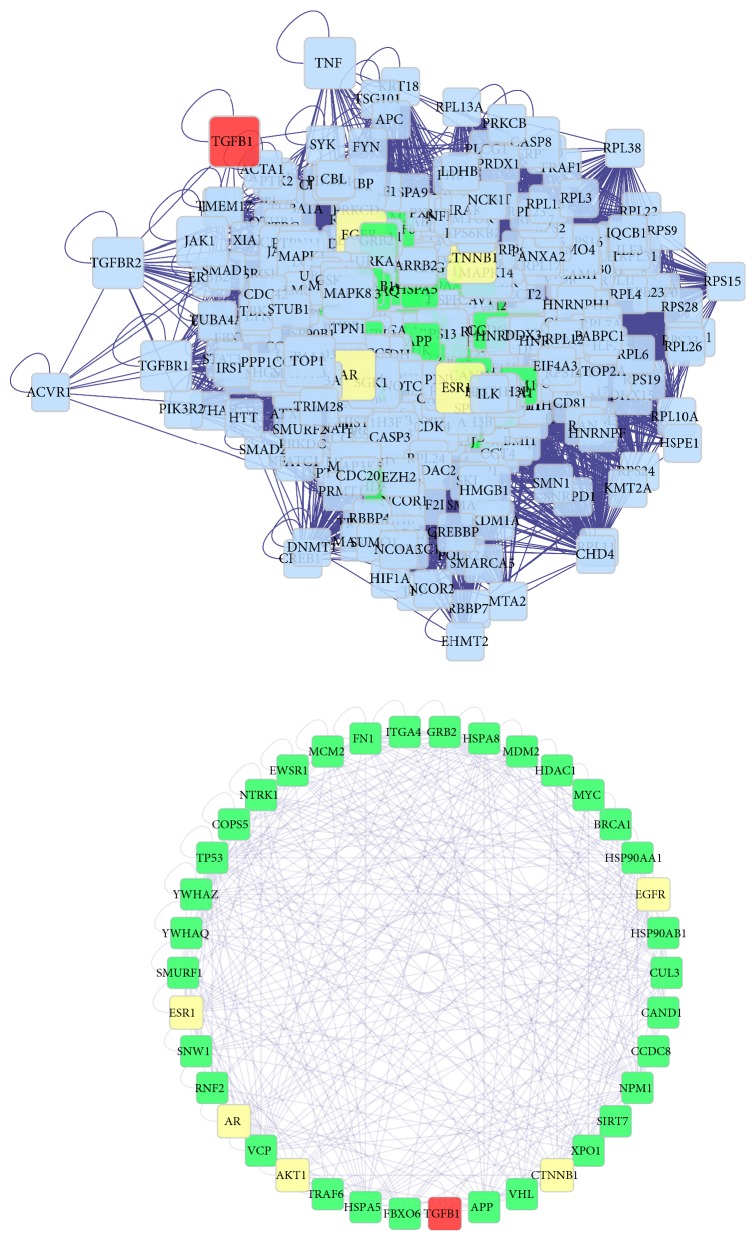
The significant compound Target-DN-Other Human Proteins' PPI Network (degree *⩾* 60, betweenness *⩾* 270.71207, and closeness *⩾* 0.41004288; yellow nodes, red nodes, green nodes, and blue nodes represent compound targets, compound targets/DN targets, and other human proteins interacting with the compound or DN targets, respectively).

We built the compound target-DN target-other human proteins' PPI network to evaluate the significance of compound targets. The network has 1626 nodes (123 compound targets, 82 DN targets, 8 compound-DN targets, and 1413 other human proteins which have a connection with compound targets or DN targets) and 36768 edges (Figure 6). The significant targets are 400 nodes, which have 13,722 edges evaluated by the three indices including degree, betweenness, and closeness (degree ≥ 60, betweenness ≥ 270.71207, and closeness ≥ 0.41004288). Finally, we selected 40 main nodes, which play an important role in the network (Figure 7). The details are described in Table S6.

**Figure 8:**
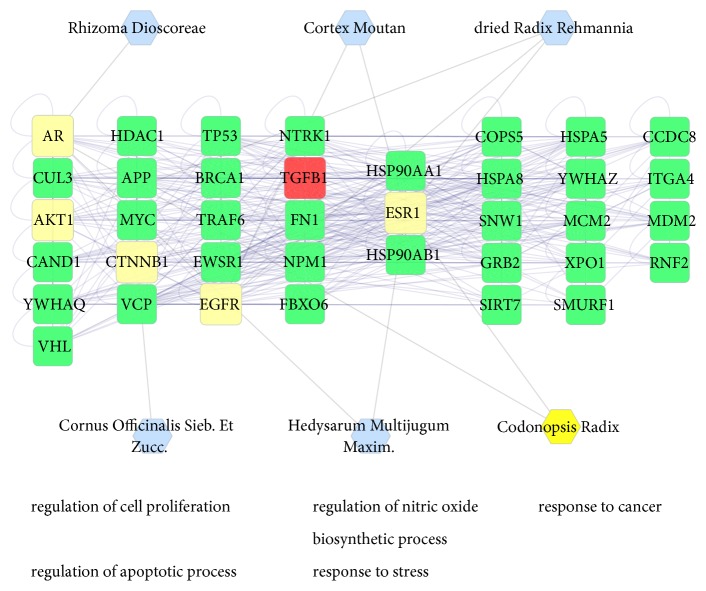
Direct interaction network between 40 major nodes in the SQDHD compound target-DN target-other human proteins' PPI network. According to the associated biological processes, compound targets of SQDHD and DN targets are related to various molecular mechanisms of DN (yellow nodes, red nodes, green nodes, and blue nodes represent compound targets, compound targets/DN targets, and other human proteins interacting with compound targets or DN targets respectively).

Furthermore, the significant compound Target-DN-Other Human Proteins' PPI Network has 400 major nodes and 13,722 edges, with the 40 main nodes include five compound targets, 1 compound target and/or DN target, and 34 other human proteins interacting with the compound or DN targets. In Figure 8, according to GO enrichment analysis of the 40 main targets, a significant connection with a negative regulation of the apoptotic process (GO: 0043066; Fold Enrichment = 8.74;*P* < 0.001), regulation of signal transduction by p53 class mediator (GO: 1901796; Fold Enrichment = 23.69;*P*< 0.001), positive regulation of nitric oxide biosynthetic process (GO: 0045429; Fold Enrichment = 48.81;*P*< 0.001), positive regulation of cell proliferation (GO: 0008284; Fold Enrichment = 8.10;*P*< 0.001), response to stress (GO: 0006950; Fold Enrichment = 27.52;*P*< 0.001), a negative regulation of the TGF-*β* receptor signaling pathway (GO: 0030512; Fold Enrichment = 26.23;*P*< 0.001), and regulation of nitric oxide synthase activity (GO: 0050999; Fold Enrichment = 48.43;*P*= 0.0016). The details are described in Table S7.

**Figure 9:**
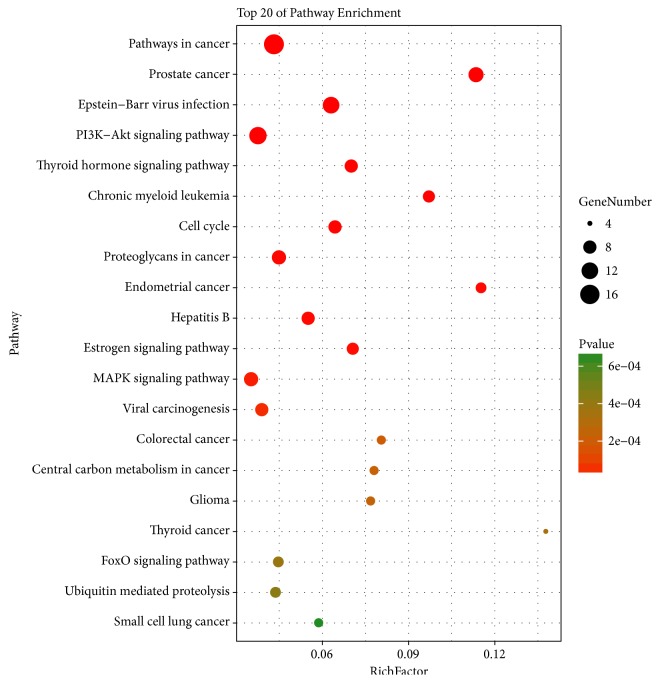
Differentially expressed gene PATHWAY enrichment point diagram of compound Target-DN-Other Human targets.

In Figure 9, according to KEGG enrichment analysis, compound targets, DN targets, and other human proteins are significantly associated with the PI3K-Akt signaling pathway, cell cycle, MAPK signaling pathway, and others. The details are described in Table S8.

The largest number of targets, biological processes, and pathways related to SQDHD and DN has been reported in some literature. Nitric oxide (NO) is one such example that has actively been associated with the kidneys through several segments consisting of the regulation of renal hemodynamics, renin secretion, inhibition of tubular sodium reabsorption, tubuloglomerular feedback (TGF), and renal sympathetic nerve activity [90, 91]. NO synthase (NOS), including neuronal NOS (nNOS or NOS1), inducible NOS (iNOS or NOS2), and endothelial NOS (eNOS or NOS3), can promote the synthesis of NO that dilates blood vessels. NOS inhibitor (L-NAME) was used for Otsuka Long-Evans Tokushima Fatty spontaneous diabetic rat models and Long-Evans Tokushima Otuska rat models as age-matched controls. As a result, the diabetic group had lower urinary NO2 + NO3 and higher urinary protein compared to the control groups. The NOS inhibitor was observed to aggravate the diabetic kidney disease [92]. Research has reported that posttranslational regulation and phosphorylation of nNOS and eNOS play vital roles in activating and inhibiting NO synthesis in the kidney [91]. Sedentary Zucker diabetic fatty (Sed-ZDF) rats had lower eNOS and nNOS expression than male ZDF rats with exercise for 8 weeks [93].

Chronic elevated blood glucose level in diabetics results in oxidative stress and inflammation associated with diabetic complications such as DN. ROS/NS overproduction caused by elevated glucose levels and enzymatic and nonenzymatic antioxidant defense deficiency can lead to oxidative stress. The influence of ROS/NS in cell signaling pathways has been linked to tissue metabolism, cell proliferation, and cell death [94]. The markers for ROS/NS damage is also seen to rise in diabetic kidneys. Research has reported that TGF-*β* in the diabetic kidney cortex is related to oxidative stress, vascular cell adhesion molecule 1 (VCAM-1), and monocyte chemotactic protein 1 (MCP-1) [9].

Several growth factors and cytokines, including TGF-*β*, VEGF, PDGF, CTGF, and others, are likely mediators of the influence of high blood glucose level on the kidneys. TGF-*β*1 has been proven as the vital cytokine that is linked to the glomerular pathology of the extracellular matrix (ECM) that is typically seen in DN. TGF-*β*1 prevents cell proliferation and apoptosis, although inducing hyperplasia and hypertrophy of mesangial cells. Diabetic animals had increased TGF-*β* in the glomerular [95]. TGF-*β*1-dependent and protein kinase C dependent pathways induce CTGF in high glucose, and CTGF may be a mediator in the process of matrix production driven by TGF-*β*1 [96]. VEGF-A, a family of secreted glycoprotein isoforms, is an endogenous protective factor and mainly produced by podocytes in glomeruli. Elevated VEGF-A protects the glomerular microvasculature in diabetics, prevents apoptosis of vascular wall cells, and prevents the development of DN [97, 98]. Targets such as FBXO6, HSPAS, HSPA5, SIRTT, and the pathway involved have shown that the SQDHD compound targets, DN target, and other human proteins have great relationship in cancer regulation, though the reported research lacks vital information. Therefore, the role of SQDHD in the regulation of cancer should be further studied.

Intensive treatment of hyperglycemia and hypertension is the primary treatment for DN. Western medicines, including ACEI and ARB, can protect renal function of DN; owing to shortage, a sufficient amount is lacking to delay or retard the progression of DN. TCM has been used in treating diabetes and preventing its complications. Hachimijiogan (HJG), consisting of* Rehmannia* radix, Fructus Corni, and others, could decrease TGF-*β*1 and iNOS levels in the kidney cortex and reduce urinary protein, serum glycosylated protein and AGEs. LDP, including* Rehmannia glutinosa*, Fructus Corni, cortex Mountain,* Dioscorea opposita*,* Poria Cocos*, and* Alisma Orientale*, decreases urinary albumin excretion rate levels.* Astragalus* (Huang Qi in Chinese) could prevent the early proliferation of mesangial cell, AGEs-mediated cell apoptosis, and reduce TGF-*β*1 expression. Moutan cortex can ameliorate inflammation via targeting RAGE* in vitro* or* in vivo*.* Rehmannia Radix (Di Huang)* can reduce TGF-*β*1, CTGF, and Ang II in high fat diet-fed plus STZ-induced diabetic rats [99, 100]. In Figure 8, according to KEGG enrichment analysis, compound target-DN target-other human proteins are significantly related to pathways in cancer, the estrogen signaling pathway, cell cycle, and, as previously mentioned, the PI3K-AKT signaling pathway, thyroid hormone signaling pathway, and MAPK signaling pathway. PI3K-AKT signaling pathway has been indicated as the source of glomerular hypertrophy and ECM accumulation [101, 102]. Some research reported that the thyroid hormone, triiodothyronine (T3), can inhibit transcriptional activation of TGF-*β*/SMAD via binding to its nuclear receptors (TRs) [103]. A reduction in the activity of the Akt signaling pathway, which is controlled by hyperglycemia, may activate p38 MAPK, inflammatory and fibrotic markers, further leading to DN [104]. P38-MAPK in vascular smooth muscle cells and aorta can be phosphorylated by hyperglycemia in diabetic rats [105].

Therefore, previous research as well as our study has shown that SQDHD can treat and prevent diabetes and its complication, including DN, via regulating several targets linked to the NO biosynthetic process, nitric oxide synthase activity, cell apoptosis, cell proliferation, and stress. Some mechanisms of SQDHD on DN covered by network pharmacology have been reported, and new mechanisms can be further verified with animal and cell experiments.

## 4. Conclusion

The TCM herbal formula, a vital type of complementary and alternative medicine, is widely used to treat diabetes mellitus and complications. The study shows that SQDHD may attenuate high glucose level, high blood pressure, oxidative stress, cell apoptosis, and proliferation related to DN, through the adjustment of its candidate targets and pathway by employing network pharmacology. Uncovering the pharmacological mechanism and demonstrating these prediction targets with experiments are required in future studies.

## Data Availability

The data used to support the findings of this study are available from the corresponding author upon request.

## Supplementary Materials

Supplementary Materials Table S1: Composite compounds of each herb in SQDHD. Table S2: Compound targets for each herb in SQDHD. Table S3: DN targets. Table S4: Enrichment analysis of compound target, DN target, and compound target/DN target based on Gene Ontology (GO) annotation. Table S5: Enrichment analysis of compound target, DN target, compound target/DN target, and other human protein based on Gene Ontology (GO) annotation. Table S6: Topological features of major nodes. Table S7: Enrichment analysis of compound target-DN-other human proteins based on Gene ontology (GO) annotation.
